# Evaluation of a Fast Test Based on Biometric Signals to Assess Mental Fatigue at the Workplace—A Pilot Study

**DOI:** 10.3390/ijerph182211891

**Published:** 2021-11-12

**Authors:** Mauricio A. Ramírez-Moreno, Patricio Carrillo-Tijerina, Milton Osiel Candela-Leal, Myriam Alanis-Espinosa, Juan Carlos Tudón-Martínez, Armando Roman-Flores, Ricardo A. Ramírez-Mendoza, Jorge de J. Lozoya-Santos

**Affiliations:** 1 Department of Mechatronics, School of Engineering and Sciences, Tecnologico de Monterrey, Monterrey 64849, Mexico; mauricio.ramirezm@tec.mx (M.A.R.-M.); patriciotijerina@hotmail.com (P.C.-T.); a01197730@itesm.mx (M.O.C.-L.); myriam.alanis@tec.mx (M.A.-E.); armando.roman@tec.mx (A.R.-F.); ricardo.ramirez@tec.mx (R.A.R.-M.); 2 School of Engineering and Technologies, Universidad de Monterrey, San Pedro Garza García 66238, Mexico; juan.tudon@udem.edu

**Keywords:** fatigue, biometrics, electroencephalography, modeling, wearable sensors

## Abstract

Non-pathological mental fatigue is a recurring, but undesirable condition among people in the fields of office work, industry, and education. This type of mental fatigue can often lead to negative outcomes, such as performance reduction and cognitive impairment in education; loss of focus and burnout syndrome in office work; and accidents leading to injuries or death in the transportation and manufacturing industries. Reliable mental fatigue assessment tools are promising in the improvement of performance, mental health and safety of students and workers, and at the same time, in the reduction of risks, accidents and the associated economic loss (e.g., medical fees and equipment reparations). The analysis of biometric (brain, cardiac, skin conductance) signals has proven to be effective in discerning different stages of mental fatigue; however, many of the reported studies in the literature involve the use of long fatigue-inducing tests and subject-specific models in their methodologies. Recent trends in the modeling of mental fatigue suggest the usage of non subject-specific (general) classifiers and a time reduction of calibration procedures and experimental setups. In this study, the evaluation of a fast and short-calibration mental fatigue assessment tool based on biometric signals and inter-subject modeling, using multiple linear regression, is presented. The proposed tool does not require fatigue-inducing tests, which allows fast setup and implementation. Electroencephalography, photopletismography, electrodermal activity, and skin temperature from 17 subjects were recorded, using an OpenBCI helmet and an Empatica E4 wristband. Correlations to self-reported mental fatigue levels (using the fatigue assessment scale) were calculated to find the best mental fatigue predictors. Three-class mental fatigue models were evaluated, and the best model obtained an accuracy of 88% using three features, β/θ (C3), and the α/θ (O2 and C3) ratios, from one minute of electroencephalography measurements. The results from this pilot study show the feasibility and potential of short-calibration procedures and inter-subject classifiers in mental fatigue modeling, and will contribute to the use of wearable devices for the development of tools oriented to the well-being of workers and students, and also in daily living activities.

## 1. Introduction

Mental fatigue refers to the effects of prolonged cognitive activity demands [1]. As the progress of fatigue continues, a decrease in vigilance and performance capacity manifests, which translates into reduced competence and willingness in maintaining and performing specific tasks [2]. This condition and its negative effects are of common occurrence across different daily-life activities and environments, such as in the workplace [3], at school [4], when driving [5] and even at some stages of physical exercise [6]. In some cases (e.g., industry workers, drivers and pilots), mental fatigue is related to many hazardous risks and accidents that represent both economic and human losses [3,7]. Therefore, reliable methods that detect this condition are highly necessary to prevent risks.

This type of fatigue is an occupational issue derived from work-related conditions, such as shift work [8], work schedule [9], and emotional distress [10]; it is further related to reduction of cognitive functions among workers, which can lead to a decrease in productivity [11]. This condition is subjective [12] and therefore, in order to properly assess it, multiple efforts have been made on translating self-assessment questionnaires [13] and harmonizing health and safety scales [14]. Alternatively, more modern, artificial intelligence (AI)–based proposals are capable of the following: improving safety in construction sites using internet of things (IoT) technology [15]; predicting injury outcomes and number of days away from work [16]; analyzing thermal protective and thermophysiological comfort performance of fabrics [17]; and protecting workers health, safety and well-being [18].

In 2019, the Mexican government published the NOM-035-STPS-2018 regulation, which obligates employers to identify, analyze and prevent anxiety and work-related stress among workers [19]. This norm is in compliance with the international norm ISO 10075-3:2004, which refers to the ergonomic principles of measurement and assessment of mental workload. The implementation of the NOM-035-STPS-2018 intends to prevent risks in the workplace and promote a safe work environment, which has a positive impact on employers’ health and well-being. However, due to the obligatory aspects of this norm, Mexican companies will need to implement reliable mental fatigue measurement protocols to ensure the mental state of their employees. Such protocols need to be easy to implement in order to be used at any time during a work shift. In this sense, the goal of this study is to propose a mental fatigue detection procedure which is relatively fast and easy to implement in different contexts.

Several reports exist in the scientific literature about mental fatigue detection by tracking changes in neural activity through electroencephalography (EEG), which is considered the ‘gold standard’ for mental fatigue assessment [20]. However, most of these studies present common methodological patterns, including the following: (1) the use of gel-based EEG caps [21,22,23], which require a considerable setup time, proportional to the number of electrodes in the cap; (2) the use of often long (>50 min) fatigue-inducing tests to identify different levels of mental fatigue during model evaluation [20,23,24,25]; and (3) the construction of predictive models which are highly subject-specific [22,26,27,28].

In a recent brain–computer interface (BCI) technology review [29], it is highlighted that one of the major challenges in the field is the long calibration time needed for some applications. They also urge to find solutions to reduce calibration periods such as the use of dry electrode EEG systems and optimized experimental designs. EEG caps with dry electrodes require on average, one third of the setup time when compared to those with gel-based electrodes, while offering similar comfort, performance and signal reliability [30]. From the revised related work, only a few studies have used mental fatigue recognition on dry electrode EEG headsets [30,31,32].

New developments in EEG technologies have started to explore the use of dry electrodes for more portable solutions [33]. Highly portable EEG headsets, such as the Thinkmindset [34], and the Muse [35] (3 and 4 dry electrodes respectively), allow to obtain fast EEG acquisitions with none or very reduced setup time. Few-channel EEG equipment are often preferred in some applications, as they are more comfortable and better suited for real-world scenario studies [36]. Some EEG studies have explored the classification of neural activity using data from a single electrode for BCI spellers [37], and motor imagery BCIs using three electrodes [38] with 97% and 81% accuracy respectively. This few-channel approach reduces the complexity and preparation time of the system, and at the same time increases its usability.

Typically, mental fatigue studies include in their protocols fatigue-inducing tests. The duration of such tests varies from one study to another. Some examples include 50 min short-term memory experiments [39], 1 hr auditory vigilance tasks (AVT) [40], 90 min simulated driving [23], 2 hr arithmetic tasks [20], and 150 min simulated driving experiments [24]. Although the use of such procedures ensures that the participants are in a fatigued state while measuring their neural activity, the time consumed is considerably large, and such protocols do not fit in our proposed fast setup scheme. Calibration-free methods to identify mental fatigue are also suggested in [41] to avoid the time-consuming process of inducing fatigue.

Due to the variability in neural activity from one user to another, many mental fatigue recognition studies implement customized EEG-based algorithms in which different classifiers are trained to a specific subject’s signals, using different machine learning and deep learning techniques [42]. Some examples of classic machine learning methods include the following: a Fisher linear discriminant analysis (FLDA) approach used to classify mental fatigue with 98% accuracy [39]; a kernel partial component analysis (KPCA)–support vector machine (SVM) approach to classify from three mental fatigue states with an accuracy of 81.64% [24]; a two-states, SVM classifier using EEG and electrocardiographic (ECG) spectral features with 91% accuracy [20]; and a five-level mental fatigue SVM achieving 91.2% accuracy [40]. In the deep learning context, algorithms such as long short-term memory (LSTM), recurrent neural networks (RNN) and convolutional neural networks (CNN) were implemented. A bidirectional LSTM (BLSTM) model was able to classify the level of drowsiness in pilots under simulated flight obtained 87% and 69% accuracies when classifying two and five drowsiness levels, respectively [22]. An RNN model with an accuracy of 92.95% for driving mental fatigue detection was presented in [23]. A two-class EEG-based mental workload estimation using BLSTM-LSTM architecture with an average accuracy of 86.33% was implemented in [43]. An EEG-based spatio-temporal CNN (ESTCNN) model showed a high accuracy of 97% when identifying between alertness and fatigue states during simulated driving tasks [26].

All the aforementioned works used a subject-specific approach in their protocols and data processing. An inter-subject transfer learning approach was followed in [42] to develop a multi-participant model for mental fatigue recognition under simulated driving tasks. Although the accuracy of the obtained model was lower than in other works (73%), the study proposes an interesting take on the development of universal classifiers and calibration-free methods. As stated in [42], general classifiers allow inter-subject classification by using information from different samples (subjects) as input, and are able to model features from multiple participants. The development of general models allow the avoidance of the time-consuming task of creating one personalized model per participant. This is highly important when using data from large databases, as one general model could be used to represent all participants without the need of several individual models.

Among the related scientific literature, EEG, ECG and other physiological signals were used as features for mental fatigue recognition. In EEG, posterior alpha (8–12 Hz) and anterior theta (4–7 Hz) waves were found to be directly related to mental fatigue [44]. The increase of theta power, as well as the theta/alpha ratio, have shown positive correlation to mental fatigue states [27]. During the condition of mental fatigue, users experience a shift from an alertness state to a more relaxed state, which is reflected in EEG as an increase in low frequency waves, and a reduction in high frequency waves [39,45].

There are also studies on time-locked event-related potentials (ERP) in EEG to observe changes in temporal aspects of brain response in fatigued users [46,47]. The P300 waveform is widely used in a BCI context, as well as for mental fatigue evaluation [48,49,50]. The P300 is a positive peak presented approximately 300 ms after the onset of a stimulus [51]. The height of this peak is larger when users are presented with an unexpected, low probability stimulus. Based on this premise, the oddball task emerged as the standard protocol to elicit P300 responses. The oddball tasks present a user two types of stimuli (frequent and non-frequent) to observe the P300 waveforms [52,53]. Two parameters of interest of the P300 wave are (1) latency, which is the time when the peak of the wave appears, and (2) its amplitude. Variations in these parameters are associated with the development of mental fatigue [54,55]; a decrease in amplitude and an increase in latency are associated with the development of mental fatigue. In such a state, the performance of cognitive tasks reduces, which reflects as a delayed stimulus identification, as well as a decreased arousal level [21]. Similar latency increases of ERPs are related to the aging process as well, as shown by auditory oddball (AO) protocols [56].

Heart rate variability (HRV) was also monitored to estimate user’s mental fatigue and drowsiness [57]. Power increases in the low frequency (LF) component of ECG ( 0.1 Hz) were observed after continuous performing of monotonous tasks [20]. It is also possible to observe the relation between sympathetic and parasympathetic activity through an analysis of heart-related signals. An increase in heart rate (HR) can be related to an increase in sympathetic activity and vice versa. Alternative HR and HRV measurements can be obtained through photopletysmography (PPG), which is an optical signal that measures blood volume changes in micro vascular tissue [58]. Other physiological measures were studied during fatigue-inducing experiments, such as electrodermal activity (EDA) and body temperature [59]. EDA measures changes in skin resistance associated with sweating; therefore, it is a sensitivity index of sympathetic nervous system activity. An increase in EDA and a decrease in body temperature were observed during sleep deprivation experiments, as well as a reduction in reaction time to presented stimuli [59]. Another study presents a CNN model which used biometric data (EDA, HR and Temperature) gathered from six weeks to predict the fatigue level of users, obtaining an accuracy of 82.9% [60].

Following the aforementioned methods in mental fatigue recognition, employers that want to comply with well-being regulations would need to ask their workers to undergo fatigue-inducing tests, and then build one specific classifier for each of them, which is not practical. Therefore, the aim of our study is to implement a fast mental fatigue detection test, able to be implemented under different work scenarios. In this sense, we propose three key components: (1) the use of a dry electrodes EEG headset, (2) the design of a short-calibration experimental protocol that does not need to induce mental fatigue, and (3) the construction of an inter-subject fatigue recognition algorithm. In this pilot study, multiple linear regression (MLR) models were used, as suggested in [27] to contribute in the development of more simple and general models. Fatigue-inducing tests are not part of this study. Instead, participants are asked to answer a self-reported fatigue questionnaire, which is used as ground truth to model the fatigue recognition algorithms. This procedure implies a very reduced setup time, ensuring the short-calibration design.

Regarding the analyzed features to build such models, our proposal uses a combination of highly portable wearables, which allow simultaneous recording of EEG, PPG, EDA and body temperature. Generalizable features are preferred in the multi-participant scheme, such as P300 parameters, power band ratios and normalized features. The equipment used is useful to implement measurement and predictive tools in real-world scenarios, such as workplace and educational scenarios. Using the proposed procedure, it was possible to design and evaluate a time-efficient and easy-to-implement mental fatigue assessment tool. Such a tool can be used in industrial, office and educational environments with the purpose of identifying the mental fatigue condition of the users. This tool could be used to infer the mental fatigue state of a user based on its biometric signals and to provide reliable feedback. The experimental setup and design is described in Section 2, while the signal processing, feature extraction and model evaluation methods are presented in Section 3.

## 2. Data

The proposed fatigue assessment tool includes the measurement of biometric data, such as EEG, HR, HRV and EDA through wearable devices (OpenBCI helmet and Empatica E4 wristband) during a five-minute recording. Biometric data are transferred to a PC to perform analysis, feature extraction and fatigue modeling to evaluate prediction of previously self-reported fatigue scores.

### 2.1. Experimental Design

A total of 17 healthy subjects volunteered in this study, with a mean age of 22 years and standard deviation of ±3 years (8 male and 9 female). Subjects were informed beforehand of the experimental procedures and were asked to sign a consent form, informing them of their right to leave the experiment if feeling uncomfortable at any moment. All participants provided signed consent for data sharing and publication.

**Figure 1:**
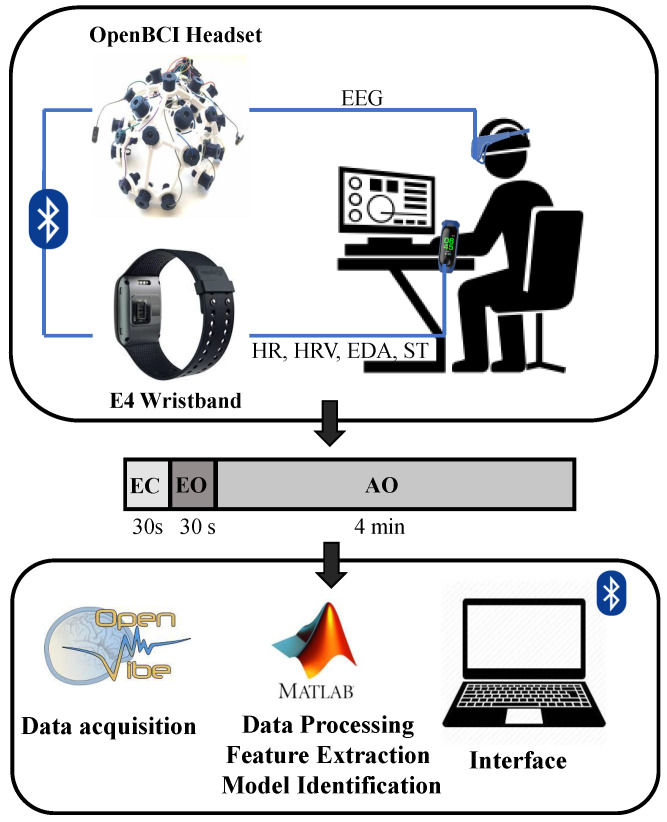
Concept for the experimental design of the fatigue assessment tool. Biometric data are acquired through the OpenBCI and E4 wearables during a relaxed state (EC and EO) and during the AO task. In the AO task, frequent and non-frequent stimuli are presented randomly in a 80:20 proportion. Data are transferred to a PC via Bluetooth, using the OpenVibe software and analyzed offline in Matlab for data processing, feature extraction and model assessment.

After explaining the procedures and obtaining the consent forms signed, users were asked to answer a fatigue questionnaire, the fatigue assessment scale (FAS) test. Then, the Ultracortex “Mark IV” EEG headset, (OpenBCI, New York, NY, USA) and the E4 wristband (Empatica, Milano, Italy) were adjusted over the user’s head and left wrist, respectively. Volunteers were asked to remain seated in a relaxed, comfortable position while measuring their physiological signals. EEG and biometric recordings were initialized in synchronization. Measurements took place in an environment replicating working settings, (e.g., classroom or office). All participants underwent a 5 min recording, consisting of a 30 s eyes closed (EC) recording, followed by a 30 s eyes open (EO) measurement, and a 4 min AO task, to elicit P300 waves. The change between EC and EO tasks was marked by a three-second low pitched tone, as well as the change between EO and AO. EC and EO were used for measurement of baseline state signals of each user, as well as preparation for the AO trials to avoid the sudden start of experiments. A conceptual framework of the proposed mental fatigue assessment tool is presented in Figure 1.

The AO task was formed by the presentation of a total of 120 auditory stimuli of two classes (two different tones): 96 frequent and 24 non-frequent stimuli, with a 80:20 probability proportion, as suggested in [53,61]. Stimuli were presented for one second, with an inter-stimulus period of one second. Volunteers were instructed to use a set of earphones during the recordings to avoid distractions from surrounding noise. Participants underwent one trial each of the explained protocol. Stimulus presentation protocols were designed using OpenVibe software, which allows real-time acquisition, filtering, processing, classifications and visualization of brain signals [62]. Data from the E4 wristband were stored in a cloud server and accessed using the E4 Manager app. MATLAB was used for offline processing of the obtained data, feature extraction and model evaluation.

### 2.2. EEG Acquisition

**Figure 2:**
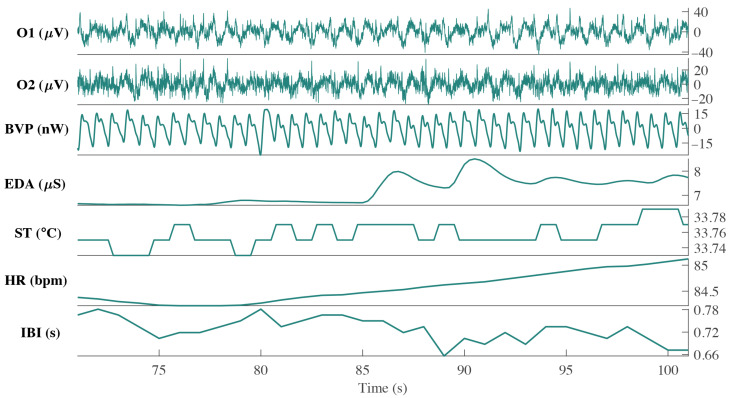
Thirty-second visualization of biometric signal acquisition during AO task for participant P ${}_{2}$ . Each row represents a different signal in the same time frame. From top to bottom: EEG (O1, O2) obtained from the OpenBCI headset, and BVP, EDA, ST, HR and IBI, obtained from the E4 wristband.

EEG signals were acquired using the wireless OpenBCI equipment along with the Cyton Board. This system allows to obtain EEG signals, using dry electrodes at a sampling frequency of 250 Hz. The Ultracortex Mark IV headset was used to set the EEG electrodes. It is an open-source, 3D-printable headset intended to work with any OpenBCI Board and allows to record EEG signals under different possible configurations. These properties allow for fast, simple implementation and set up during the experiments [63]. This systems makes use of eight EEG channels of the standard 10–20 system: FP2, FP1, C4, C3, P8, P7, O1, and O2, and two clip reference electrodes (one placed in each earlobe). Using OpenViBE, signals were further filtered using a 60 Hz Notch filter to remove powerline noise, and 0.1 –100 Hz 4th order Butterworth bandpass filter, as brain activity lies within this frequency range [64]. The first two rows of Figure 2 show a 30 s visualization of EEG acquisition of a representative participant on two electrodes (O1 and O2).

### 2.3. Biometric Signals Acquisition

The Empatica E4 wristband is a wireless wearable system that measures biometric signals in real time [65]. This system measures PPG, inter-beat interval (IBI), HR, EDA and skin temperature (ST) signals. Signals were obtained at 64 Hz (PPG), 4 Hz (EDA and ST) and 1 Hz (HR and IBI). The last five rows of Figure 2 show a 30 s visualization of signals (BVP, EDA, ST, HR and IBI) of one representative participant.

### 2.4. Fatigue Assessment Scale

Mental fatigue level was measured for all volunteers, using the score obtained in the self-answered FAS questionnaire. The FAS is a tool which represents a valuable instrument for fatigue assessment with consistent reliability and validity [66], and has been validated in several studies in the literature [67,68,69]. In [67], the use of the FAS was used for the identification of mental fatigue in young adults. Furthermore, the questions from the FAS, as stated in [69], were selected from four previous valid questionnaires: the fatigue scale (FS), the checklist individual strength (CIS), the emotional exhaustion subscale of the Dutch version of the Maslach Burnout Inventory (MBI-DV), and the Energy and Fatigue Subscale of the World Health Organization Quality of Life assessment (WHOQOL) instrument.

Due to the fact that Spanish was the first language of all volunteers, the Spanish version of the FAS was used in this study [70]. The FAS questionnaire consists of 10 questions with an individual score (1–5). The questions and possible answers from the FAS are presented in Figure S1 (see Supplemental Material). According to [71], the FAS total score can be classified into three classes: no fatigue (1–21), substantial fatigue (22–35) and extreme fatigue (36–50). From the 17 participants, 3 were in the no fatigue class (19 ± 2), 8 in substantial fatigue (27 ± 3), and 6 (37 ± 3) in extreme fatigue. A distribution plot of the FAS scores of the 17 participants is presented in Figure S2 (see Supplemental Material).

## 3. Methods

**Figure 3:**
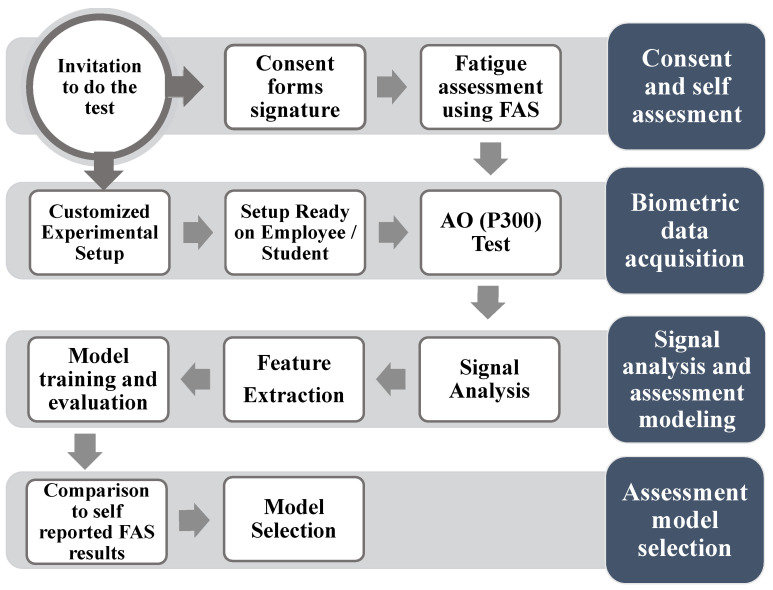
Diagram of the methodology implemented in the study. After data acquisition, the signal processing, feature extraction and model evaluation steps were performed.

The methods used in this study are mainly divided into the following: signal acquisition and processing, feature extraction, and model training and evaluation. A simplified diagram of the methodology is presented in Figure 3.

### 3.1. EEG Temporal (P300) Analysis

EEG signals were pre-processed prior to analysis to reduce unwanted noise and remove artifacts. Besides the bandpass filters mentioned in Section 2.2, all EEG signals were further cleaned using the artifact subspace reconstruction (ASR) algorithm, using a parameter κ=15 to reduce large artifacts. The ASR is an effective and efficient signal cleaning method that reconstructs artifacts as large as κ -times the standard deviation of a clean portion of the signal [72]. Values of κ between 10 and 100 are recommended for optimal ASR filtering, according to [72]. The choice of the parameter κ was selected, as, by using this value, the artifact filtering is not as aggressive as removing important EEG-related activity, but is effective at removing muscle and eye movement–related activity instead [72]. This algorithm was implemented using MATLAB’s EEGLAB toolbox [73].

P300 waves were analyzed according to the protocol described in [21]. EEG signals were divided into $N_{nf}$ 1-second windows, where $N_{nf}=24$ is the number of non-frequent stimuli in each trial. All windows were formed by 1000 ms containing the signals comprised of the 200 ms prior to the onset of the stimuli to the 800 ms after the stimuli. All windows were filtered from 0.1 –10 Hz, averaged and baseline (EO) corrected to obtain eight (one per channel) P300 representative waves per trial. To ensure that there was no phase distortion in the 1000 ms windows due to temporal filtering, a zero-phase digital filter was implemented using the MATLAB command *filtfilt*, which ensures zero phase distortion at its output.

P300 amplitudes and latencies were obtained for all EEG channels and users. The amplitude of P300 is defined as the highest positive peak of the obtained waveform in the immediate post stimulus 200–500 ms window [21], while latency is defined as the time from stimulus onset to the presentation of the aforementioned peak [74]. Typical P300 analysis involves individualized analysis per subject; however, in our inter-subject proposal, following the parameter detection guidelines reported in [21], an automated P300 latency and amplitude detection algorithm was implemented for an efficient feature selection process. Automatic P300 parameter detection was previously reported and validated in the literature [75]. The calculated amplitude and latency values obtained by these procedure were used as features (2 × 8 channels =16 features) for the MLR models.

**Figure 4:**
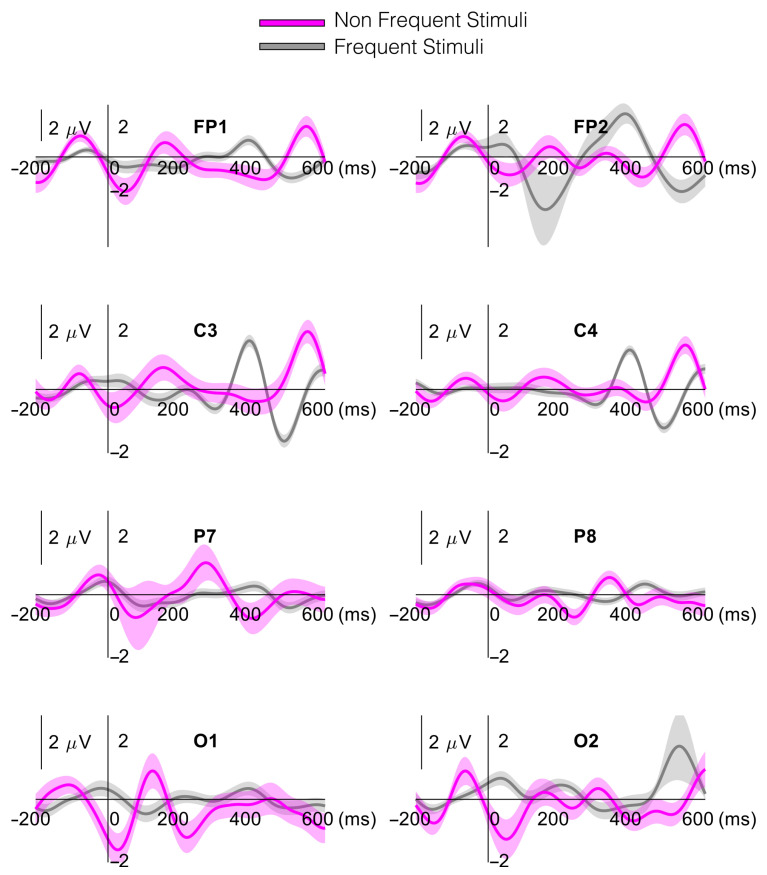
Grand average representation of the P300 wave across all participants. Eight traces (one per channel) are presented for frontal (F), central (C), parietal (P) and occipital (O) electrodes of the left and right hemispheres of the brain. Shaded area represents standard error across traces.

Figure 4 shows a P300 waveform representation obtained from the average of all subjects for both frequent and non-frequent stimuli during the AO trial at eight channels. In this representation, the P300 wave is observed around 200 ms after stimulus onset at FP1 and C3, and more prominently at 300 ms in P7.

### 3.2. EEG Spectral Analysis

EEG signals were used to calculate power in five frequency bands: Delta (1–4 Hz), Theta (4–7 Hz), Alpha (8–12 Hz), Beta (13–29 Hz) and Gamma (30–50 Hz). Power was calculated on 1-s windows using the fast Fourier transform (FFT) for all EC, EO and AO tasks in all EEG channels and frequency bands. Normalized power values (with respect to EO task) ( NP(t) ) at every 1 s window *t* were obtained for each user independently, following Equation (1).

(1) $$NP{\left(t\right)}_{ch,fb}=\frac{AO{\left(t\right)}_{ch,fb}-{\bar{EO}}_{ch,fb}}{{\bar{EO}}_{ch,fb}},$$

where ${\bar{EO}}_{ch,fb}$ represent the average power at specific channel ( ch ) and frequency band ( fb ) during the EO task, and $AO{\left(t\right)}_{ch,fb}$ represent the power values at the same channel and frequency band during the AO task before normalization. Average normalized power values for all EEG channels and frequency bands were used as features (5 frequency bands × 8 channels =40 features) for the MLR models.

Power ratios were also calculated for all possible combinations of frequency bands. A total of 20 power ratios were obtained for all EEG channels, using the estimated power values prior to normalization ( $AO{\left(t\right)}_{ch,fb}$ ). Then, average power ratios were used as features (20 ratios × 8 channels =160 features) for the MLR models. All the obtained power ratios are shown in Table 1.

### 3.3. Empatica E4 Analysis

Features from the Empatica E4 wristband were also calculated, providing information about physiological variables of each participant. Power values from PPG signals were calculated in four frequency bands: total power (TP: 0– 0.4 Hz), high frequency (HF: 0.15 – 0.4 Hz), low frequency (LF: 0.04 – 0.15 Hz) and very low frequency (VLF: 0– 0.03 Hz) [20,76]. Normalized PPG power values (with respect to EO task) were obtained using 30 s windows, on one-second moving windows for all frequency bands in a similar manner as in Equation (1). This window size was selected to correctly estimate all frequency components mentioned previously. Additionally, two ratios were calculated: the LF/HF ratio, and the LF in normalized units (LFNU). The LF/HF ratio was obtained by dividing the LF and HF features prior to normalization, and the LFNU was estimated as defined in [20] using Equation (2).

(2) $$LFNU{\left(t\right)}_{AO}=\frac{LF{\left(t\right)}_{AO}}{TP{\left(t\right)}_{AO}-VLF{\left(t\right)}_{AO}},$$

where $LF/HF{\left(t\right)}_{AO}$ represents the ratio between the LF and HF components during the AO tasks, and $LFNU{\left(t\right)}_{AO}$ represents the LF component in normalized units at each time window. Ten signals were obtained from the E4 wristband per second: VLF, LF, HF, TP, LF/HF, LFNU, IBI, HR, EDA and ST during AO trials. The average values of the calculated signals were used as features for the MLR models.

### 3.4. Feature Selection

A total of 226 features were obtained as variables for the MLR model: 16 (P300) + 40 (Normalized EEG Power) + 160 (EEG Power Ratios) + 10 (E4). Signals from three subjects were discarded due to missing data. Therefore, the presented results take into account the remaining 14 participants. Correlational analyses were extensively used in the literature to find physiological features relevant to mental fatigue [77]; therefore, the correlation between features and FAS scores was obtained.

Correlation coefficients, using the *corrcoef* MATLAB function, were calculated as well as the corresponding p-values for the correlation tests between each of the 226 features and the FAS scores, with a sample size of m=14 (participants). Features which showed significant correlation to FAS scores (*p*-value < 0.05 ) were considered in the subsequent analysis; features which did not show this condition were excluded.

The selected significant features from the correlational analysis were used to build a feature matrix, containing those features most correlated to FAS scores from all volunteers. This feature matrix was sorted according to their p-values, e.g., the first feature in the matrix, (with the lowest *p*-value), was the most correlated to FAS. The sorted feature matrix was then used for model evaluation purposes.

### 3.5. Model Training and Evaluation

Models were evaluated using three (training:testing) data splitting approaches: 70:30, 80:20 and leave-one-out (LOO), respectively. The 70:30 ratio is commonly used in machine learning approaches in EEG-based classification [78,79], and the 80:20 ratio [20] and the LOO approach [42] were implemented in similar studies. In the 70:30 ratio, a training set was built by randomly selecting data from 10 subjects and testing on the remaining 4 subjects; in the 80:20, data from 11 subjects were used for training, and 3 for testing; and in the LOO approach, the training set used 13 and 1 subjects for training and testing, respectively. In order to evaluate the performance of the predictive models, in all data splits implemented, data from participants in the training set were not included in the test set.

Models were trained and evaluated using two nested for loops. The first loop increased the number of features of the training set from 1 to K (using the sorted feature matrix), where *K* is the maximum number of significantly correlated features (e.g., the model first evaluates the most correlated feature), and the second loop enabled the random selection of a different training/testing set at every iteration (cross-validation).

This process was iterated ten times to achieve a ten-fold cross-validation (CV) to obtain an average representation of the model’s performance. In each CV, the parameters of the MLR models for each selected training set were calculated, using the normal equation method [80,81], and were used to predict the FAS scores in each test set. Following this procedure, by averaging across the 10 CVs, the performance of the models using different number of features were obtained.

Two metrics were used to evaluate model’s performance: the root mean square error (RMSE), and the average percentage of correct classification. The RMSE was used to estimate model performance under a linear regression approach, and shows the difference between the predicted and real FAS scores. When evaluating RMSE, it is desirable to be as minimized as possible to increase the model’s performance. The RMSE was obtained using the following:

(3) $$RMSE=\sqrt{\sum_{i=1}^{n}\frac{{\left(Y_{i}-\hat{Y_{i}}\right)}^{2}}{n}},$$

where $Y_{i}$ and $\hat{Y_{i}}$ are the real and predicted FAS scores, respectively, and *n*, the number of examples in the evaluated test set. The average RMSE was obtained across CVS.

To evaluate the performance of the models under a classification approach, the accuracy of the models was calculated as the average percentage of correct classifications across CVs. In this case, the model is evaluated on how well it predicts the correct classes of the FAS questionnaires answered by the participants.

Three MLR models were designed and evaluated to predict FAS score using different combinations of features and data splitting approaches. The first model (EEG) considered all the selected features from EEG analysis. The second model (NR-EEG) considered only the non-redundant significant features from only EEG analysis. Redundant features were removed from the analysis. For instance, if a pair of features, such as α/θ and θ/α , at the same EEG channel were significant, the feature with the higher p-value was removed from the model. The third model (E4) considered the six E4 features shown in Table 2.

The prediction performance and RMSEs of all the models were evaluated and compared, using different amounts of data from the AO task—4 min, 2 min, 1 min, 30 s and 15 s—to observe the best time period for assessing fatigue in this approach. Feature extraction, selection and model evaluation were implemented by custom codes in MATLAB 2020a version 9.8.0.1396136 (The MathWorks Inc).

## 4. Results

A total of 51 features, all of them obtained from the EEG analysis, were found to be significantly correlated to the FAS score and were included in the models. No features from the E4 analysis were found to be significant, and therefore, were discarded from the subsequent analyses. All the selected features in these analyses are shown in Table 2 with their respective *p*-values and correlation coefficients (r). For completeness, the six most correlated E4 features (ST, LF, TP, HF, LFN and LF/HF) to the FAS score are also listed.

**Figure 5:**
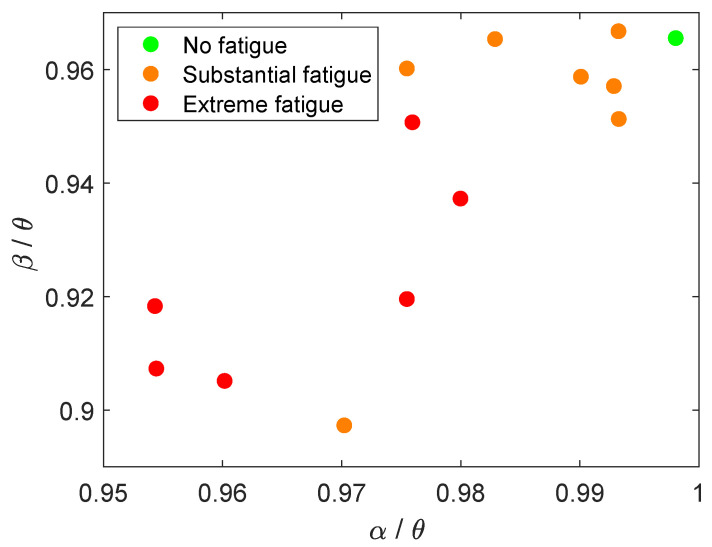
Two-dimensional representation of the most significantly correlated feature to FAS score: β/θ (C3), and α/θ (O2) in relation to their fatigue classifications.

Among the most significant features from the EEG analysis were the β/θ in C3 ( p=0.003 ) and the α/θ in the O2 ( p=0.0011 ) ratios. Figure 5 shows the calculated α/θ power ratio at electrode (O2), in relation to the β/θ ratio in C3 at different fatigue states. The fatigue state defined by the FAS score is shown in color code for the three fatigue states. Figure 5 represents a prominent negative correlation between both features and the user’s fatigue scores. Regarding the ERP analysis, the latency showed a significant correlation in C3 ( p=0.0268 ) and in P7 ( p=0.029 ), as presented in Table 2.

**Figure 6:**
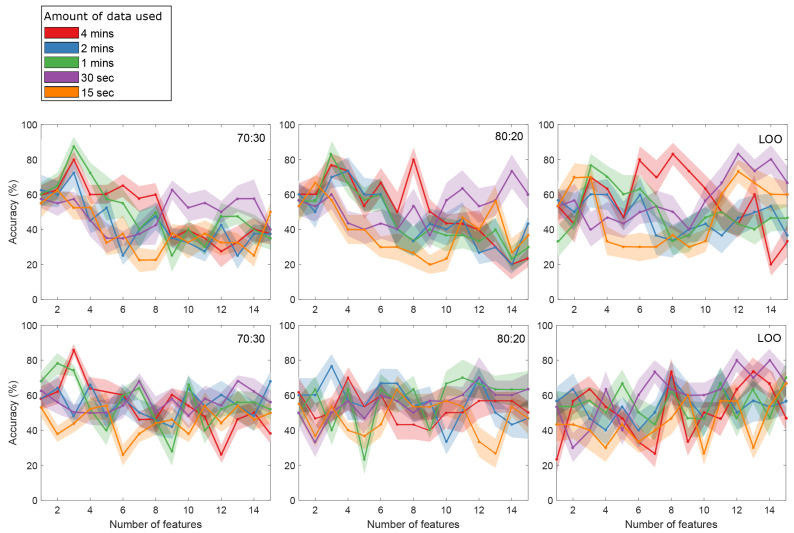
Average classification accuracies for the EEG (**top** row) and NR-EEG models (**bottom** row) ( K=15 features), using 4 min, 2 min, 1 min, 30 s and 15 s of data during the AO task. Shaded area represents standard error across cross-validations. Model performance was evaluated using 70:30 (**left**), 80:20 (**middle**) and LOO (**right**) data splitting approaches.

Figure 6 shows the average accuracy of the evaluated models across CVs from the first 15 features, using different amounts of data of the AO task recording: 4 min, 2 min, 1 min, 30 s and 15 s. In the EEG model (70:30), maximum accuracy ( 88% ) was obtained from one minute using the three most significant features: β/θ (C3), θ/β (C3), and α/θ (O2). Further feature inclusion resulted in lower accuracies. In the 80:20 model, a similar behavior to the 70:30 model was observed. In this case, 83% accuracy was the maximum using one minute of data. In the LOO model, the three feature classifier decreases its performance, and the maximum accuracy remains at 83% . However, a shift toward a higher number of features is observed: using 8 features of the four minutes recording and 12 features of 30 s of data.

The NR-EEG classifier shows, on average, lower performance than the EEG model. In this model, maximum accuracy ( 86% ) was obtained using four minutes of the three most significant features: β/θ (C3), α/θ (O2) and α/θ (C3) for the 70:30 ratio. In the 80:20 ratio, a maximum accuracy of 77% results from using three features on two minutes of data. In the LOO approach, the highest accuracy ( 80% ) was found using 12 features using 30 s of data.

**Figure 7:**
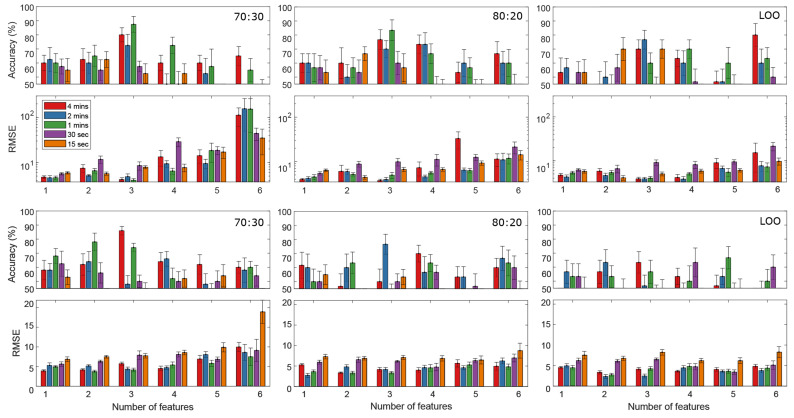
Average classification accuracy and RMSE for the EEG (**top** row) and NR-EEG models (**bottom** row), for the six most significant features. Accuracy and RMSE were calculated using different amount of data from the AO task (4 min to 15 s). Error bars represent standard error across cross-validations. Model performance was evaluated using 70:30 (**left**), 80:20 (**middle**) and LOO (**right**) data splitting approaches.

The accuracies and RMSEs from both EEG and NR-EEG models in the three data ratios are presented in Figure 7, using different amounts of data points in the AO tasks. It is observed that the highest accuracy classifier ( 88% ) also presents the lowest RMSE value of 4.058 . In the 80:20 and LOO approaches, in general for the first six features, the accuracies are lower and the errors higher than in the most accurate 70:30 model.

A slight accuracy reduction is observed in Figure 7 for the NR-EEG model, when compared to the EEG models. The highest accuracy NR-EEG classifier ( 86% ) shows a relatively low RMSE error of 5.729 . In the 80:20 and LOO approaches, it can be observed that the errors of some models are slightly smaller than in the 70:30 ratio, and some are even smaller than those in the EEG models (especially when including more features); however, the accuracies are also lower and do not surpass the best model in the 70:30 ratio.

Figure S3 (see Supplemental Material) shows the accuracy of the E4 models for all data-splitting approaches. It can be observed from these figures that such models are not precise enough for the desired application, tending to present a similar behavior across different number of features, and average accuracies of 55% and 60% for the 70:30 and 80:20 splits, respectively. The LOO approach obtained slightly higher performance, especially using 30 s of the first three features ( 80% ).

## 5. Discussion

### 5.1. Feature Analysis

In the present study, different physiological variables (EEG, BVP, EDA, temperature) were measured in a fast, short-calibration and simple-to-implement mental fatigue evaluation test, using wearable devices (portable, dry-electrode EEG and smart wristband). The advantages of the proposed tool, compared to others reported in the literature, lie in the design of the test: using dry-electrode EEG, which has a faster setup than gel-electrode EEG; there is no implementation of long fatigue-inducing tests on the participants; and the test follows an inter-subject approach.

In order to find relation between the measured variables and the mental fatigue states of the participants, pair-wise correlations between features of physiological signals and self-reported fatigue measurements were calculated in an inter-subject design. The most significant correlations to FAS score, as shown in Table 2, were mainly EEG power ratios between high and low frequency bands at central and occipital electrodes.

P300 latency was also significantly correlated to the FAS score in electrode C3, as shown in Table 2. These observed changes in latency were probably due to the topographical proximity of the motor cortex to the temporal lobes, associated to the auditory processing related to the AO tasks. The positive correlation between latency and FAS score can be interpreted as a linked increase in latency and FAS score, which reflects the delayed cognitive information processing upon the development of mental fatigue [82].

As reported in previous ERP studies, there exists a relationship between P300 features and EEG spectral changes. More specifically, a decrease in P300 amplitude correlates to a decrease in alpha power—in other words, a decrease in alpha power during a fatigued state [74,83]. In our case, a decrease in α/θ ratio was observed to correlate to a fatigued state, as observed in Figure 5. Both cases suggest that higher alpha power values are related to less-fatigued states. Other power ratios were found significant in this study, such as β/δ , β/θ , γ/δ , γ/θ , among others (See Table 2). This suggests that the ratio between the high frequency bands related to attention and alertness (beta and gamma) and low frequency bands related to drowsiness and sleep (theta and delta) is useful at determining the mental fatigue level of participants.

Physiological signals from the E4 wristband failed to prove significance to statistical tests, although they showed an apparent correlation by visual inspection. The most correlated features were ST and LF, which showed negative and positive correlations to the FAS score, respectively. A decrease in body temperature associated to fatigue development was also reported in [59]. In [20], increased LF power of ECG measurements was reported after performing fatigue inducing tasks. The authors explain that lower heart rate causes more variations in heart rate and this is reflected as an increase in the LF component [20]. In [84], a set of physiological measures (temperature, brain and heart activity) is recorded on workers, and such measures are related to fatigue. Mental fatigue signs were not observed in the study, but changes in temperature were associated to physical fatigue. In that particular study, thermoregulation was better suited to classify physical fatigue than heart rate. It could be possible that a similar phenomena happened in our case. Probably, changes observed in ST were related to physical fatigue and thus, showed a slightly higher correlation and lower p-values to FAS scores than heart-related features, which showed higher p-values. Although physical fatigue was not analyzed in this study, studies have addressed the idea of a link between mental and physical fatigue [1,6]. In [6], the development of mental fatigue in cyclists during a 20 km cycling time trial showed a negative impact on their pace regulation and overall performance.

### 5.2. Model Evaluation

Regarding the MLR models, accuracies of 88% and 86% were achieved, using one minute of AO task data for the EEG and the NR-EEG models, respectively, using a 70:30 ratio. In both cases, the features that best represent our proposed fatigue assessment model were β/θ (C3), and α/θ (O2). As the models considered more features, lower accuracies (around 60% ) were found, probably as the models were over-fitting at this point. An increase in accuracy can be observed in Figure 6, when the LOO model used 12 features, in both EEG and NR-EEG models. However, a higher amount of features in the model can lead to over-fitting and is very susceptible to noise [85]. In fact, in these cases, it is suggested to reduce the number of features and use simpler models to minimize performance reduction caused by noise [85]. Another possible solution to increase accuracy could be regularization techniques such as the least absolute shrinkage and selection operator (LASSO); however, this approach was not explored in this study. BVP, HR, IBI, EDA and ST features failed to show significance; however, the difference between the number of analyzed features from the OpenBCI and the E4 was considerably different. It is not discarded that an increase in the number of analyzed E4 features could help to increase model’s accuracy and provide a better insight on fatigue assessment.

A possible explanation on why the best model was obtained using data from the first minute of the AO task is that by the end of the task, users could be in a more relaxed state than when they answered the FAS questionnaire. Following this logic, the prediction of the self-reported fatigue state would have more sense at initial stages of the AO task, closer to the time when users answered the FAS questionnaire.

### 5.3. Limitations

Some limitations of this work that need to be addressed include the small sample size, the experimental design of the AO task, and the class imbalance of self-reported mental fatigue levels. A bigger and more balanced sample will help to increase the statistical confidence of the models to obtain higher performance. It is also known that some physiological features undergo changes due to the aging process [86]; therefore, it is important to consider that the presented work is a pilot study, and is currently being developed for the analysis of biometric signals of young adults. However, the same methods are still applicable to users in a wide range of age if an age-specific database is gathered and the models are built, for instance, for the elderly population, or middle-aged adults.

Regarding class imbalance, the major drawback is that data from two of the three participants in the no-fatigue class were excluded as mentioned in Section 3.4 due to missing data. A bigger sample size could compensate this class disparity, and increase the model’s performance. Although class imbalance is often a problem in classification accuracy, it is not a major complication in our study when observed from the linear regression approach. In the case of this type of model, the RMSE (Equation (3)) provides a notion of the performance of the model, and in Figure 7, it is observed that the most accurate model presents very small RMSE values. It is important to address that other studies in this field have reported mental fatigue, drowsiness, or mental workload predictive models, using data from a similar (and even smaller) sample size: 6 [60], 8 [6,26], 10 [22,23,40,87], 11 [42], 12 [5], 13 [24], 14 [67], and 16 [20], to mention a few. Considering the sample sizes of similar studies reported in the identification of mental fatigue, it can be discussed that the proposed pilot study holds its validity. However, the authors are aware that in future research, the experimental database should be expanded.

As presented in Table 2, P300 latency was found significant to the FAS score; however, the design of the AO task was made in such manner that stimuli were presented in a fixed inter-stimuli period. Variations in inter-stimuli can help to increase stimuli unpredictability in order to elicit more notorious P300 responses [88]. However, the P300 measurement did not seem to contribute much to the prediction of fatigue in our models, as other features were found to be more strongly correlated. Additionally, the reliability of the P300 waves increases as the number of averaged traces increases; therefore, its analysis is not very suitable for the analysis of shorter time periods. However, its parameters helped to corroborate the fatigue states of users to the FAS scores and other features.

Another limitation is the selection of a simple algorithm (MLR) to develop the predictive models. Other, more complex algorithms, were able to classify mental fatigue states with higher accuracies [26,27]. It is of our interest to implement the proposed experimental design using more complex algorithms to increase accuracy. However, our model offers some useful advantages, such as a short-calibration mental fatigue detection, a fast setup using a reduced number of dry electrodes, and generalizability across participants. As mentioned before, across this manuscript, typical procedures in mental fatigue recognition include fatigue-inducing experiments prior to model construction to ensure different fatigue states.

### 5.4. Final Remarks

In this study, mental fatigue was not induced in the volunteers, which makes the methodology closer to a real-world setting, where the protocol can be applied in a worker any time during the work shift, in a driver prior to a trip, or in a student before, during, or after a class. Subsequently, using the system on different scenarios, for instance, varying fatigue-related variables—such as shift work, work schedule, or emotional distress—the system could be able to identify the work-related condition which contributes the most to the development of mental fatigue [11]. Successfully implementing these check-ups in the business’ everyday workflow could certainly improve workers’ performance and avoid accidents, injuries, and errors. In our case, the analysis of one minute EEG data during AO task was sufficient enough to obtain an 88% accurate prediction of self-reported fatigue levels. Other important remarks are that the proposed experimental design allows a fast setup, and the models are able to generalize across subjects.

## 6. Conclusions

The described protocols allowed an easy-to-deploy five-minute test to assess non-pathological mental fatigue during work. The results presented in this study suggest that EEG features are good predictors of the FAS score, even without the need of fatigue-inducing tests, and that an 88% accuracy can be obtained from a linear model, using three features of a one-minute EEG recording. The inclusion of such biometric features in a MLR model is capable of providing a reliable fatigue assessment tool, which is free from subjectiveness [12] and cultural biases. The tool could then develop into a crucial device that properly assesses the urgency for an intervention on hazard-exposed workers, which reduce fatigue-related hazards within copious work environments [18].

Several EEG features were found to be correlated to different states of fatigue, and a more complete analysis of vital sign parameters is needed in order to improve the accuracy of the predictive models. It is also necessary to implement and test more complex algorithms under the same short-calibration design. From all features, the most useful ones to predict the FAS score of users are power ratios between high- and low-frequency bands, which represent the trade-off of increased and decreased cognitive processing capability at the moment of measurement [89].

The presented methods are implemented as an offline classification model, but could be adapted to an online classifier. In this online version, by a prior identification of relevant features on a trained model, real-time predictions can be obtained and presented to a user for biofeedback purposes. In this approach, two ’real-time’ computations would be needed to provide the predictions: feature calculation (PSD, power ratios), and a multiplication of the feature vector, and the weight vector of the trained model. The estimated time of execution to perform such computations, using the most accurate model, is 0.26 ms (see Figure S4).

The same procedures can be applied to biofeedback systems oriented to the detection of pathological mental fatigue, and provide support in therapies. The test presented in this study offers a simple solution to evaluate mental fatigue under different scenarios at the workplace, and could be useful in the upcoming Mexican government regulations on industries for workers’ well-being monitoring.

## Figures and Tables

**Table 1:** Power ratios calculated for all frequency bands.

δ	θ	α	β	γ
δ/θ	θ/δ	α/δ	β/δ	γ/δ
δ/α	θ/α	α/θ	β/θ	γ/θ
δ/β	θ/β	α/β	β/α	γ/α
δ/γ	θ/γ	α/γ	β/γ	γ/β

**Table 2:** EEG and E4 features which showed significant correlation to FAS score.

Feature	*p*-Value	r	Feature	*p*-Value	r	Feature	*p*-Value	r	Feature	*p*-Value	r
β/θ (C3)	0.0003	−0.8252	δ/β (O1)	0.0145	0.6357	Latency (P7)	0.0290	0.5819	θ/γ (FP1)	0.0438	0.5451
θ/β (C3)	0.0003	0.8242	α/δ (O1)	0.0148	−0.6343	β/δ (FP1)	0.0299	−0.5794	α (C4)	0.0441	−0.5444
α/θ (O2)	0.0011	−0.7777	γ/θ (C3)	0.0157	−0.6299	γ/β (P8)	0.0305	−0.5775	δ (C4)	0.0451	−0.5422
θ/α (O2)	0.0011	0.7760	β/δ (O2)	0.0160	−0.6287	α/δ (C3)	0.0320	−0.5734	θ/γ (O1)	0.0467	0.5390
θ/α (C3)	0.0059	0.6941	δ/α (O1)	0.0166	0.6262	γ/δ (C3)	0.0335	−0.5694	δ/γ (C3)	0.0477	0.5368
α/θ (C3)	0.0060	−0.6927	θ/γ (O2)	0.0172	0.6235	θ (C4)	0.0342	−0.5677	α/δ (FP1)	0.0487	−0.5350
γ/δ (O1)	0.0064	−0.6894	δ/γ (O2)	0.0172	0.6235	α/θ (O1)	0.0358	−0.5636	ST (E4)	0.0785	−0.5267
δ/γ (O1)	0.0071	0.6829	γ/δ (FP1)	0.0177	−0.6213	γ/θ (O1)	0.0373	−0.5599	LF (E4)	0.0869	0.5146
γ/β (O1)	0.0084	−0.6727	δ/β (O2)	0.0182	0.6193	θ/α (O1)	0.0374	0.5598	TP (E4)	0.1221	0.4711
β/γ (O1)	0.0086	0.67091	β/δ (C3)	0.0204	−0.6106	γ (FP1)	0.0383	−0.5575	HF (E4)	0.1696	0.4239
β/θ (O2)	0.0011	−0.6545	θ/γ (C3)	0.0205	0.6101	δ/β (FP1)	0.0384	0.5572	LFN (E4)	0.1902	0.4061
θ/β (O2)	0.0128	0.6445	δ/γ (FP1)	0.0211	0.6080	γ/θ (FP1)	0.0387	−0.5566	LF/HF (E4)	0.2121	0.3884
β/δ (O1)	0.0139	−0.6389	β/γ (P8)	0.0268	0.5886	δ/α (C3)	0.0400	0.5536			
γ/δ (O2)	0.0140	−0.6386	Latency (C3)	0.0268	0.5884	α/δ (O2)	0.0403	−0.5529			
γ/θ (O2)	0.0145	−0.6358	δ/β (C3)	0.0285	0.5833	δ/α (O2)	0.0429	0.54711			

## Data Availability

A dataset supporting this research is publicly available at https://ieee-dataport.org/documents/eeg-and-empatica-e4-signals-five-minute-p300-test-and-fas-scores (accessed on 5 September 2021).

## Supplementary Materials

The following are available online at https://www.mdpi.com/1660-4601/18/22/11891/s1. Figure S1: Questions conforming the self reported FAS questionnaire, and the possible answers with their individual scores. The total FAS score is obtained by summing the scores of each question (note that questions 4 and 10 must be inversely scored), Figure S2: Distribution of FAS Scores obtained for all 17 participants during the FatigueAssessment stage, Figure S3: Average classification accuracies (top) and F-Scores (bottom) for the E4 model (K = 6 features) using 4, 2, 1 (min), 30 and 15 (s) of data during the AO task. Shaded area represents standard error across cross-validations. Model performance was evaluated using 70:30 (left), 80:20 (middle), and LOO (right) data splitting approaches, Figure S4: Average computation time needed to real-time make predictions using the one-minute (most accurate) EEG model. One hundred simulations were run, and the shaded area represents the standard error across the simulations.
