# Supplementary material for: Evaluation of a Fast Test Based on Biometric Signals to Assess Mental Fatigue at the Workplace—A Pilot Study
Source: Int J Environ Res Public Health. 2021 Nov 12;18(22):11891. doi: 10.3390/ijerph182211891 (PMC8621458; doi:10.3390/ijerph182211891)
Supplement: Supplementary file 1 [file ijerph-18-11891-s001.zip › ijerph-1389272-supplementary.pdf]

Questions	Possible answers with individual scores				
	(1) Never	(2) Sometimes	(3) Regularly	(4) Often	(5) Always
1. I am bothered by fatigue					
2. I get tired very quickly					
3. I don’t do much during the day					
4. I have enough energy for everyday life					
5. Physically, I feel exhausted					
6. I have problems to start things					
7. I have problems to think clearly					
8. I feel no desire to do anything					
9. Mentally, I feel exhausted					
10. When I am doing something, I can concentrate quite well					

Figure S1. Questions conforming the self-reported FAS questionnaire, and the possible answers with their individual scores. The total FAS score is obtained by summing the scores of each question (note that questions 4 and 10 must be inversely scored).

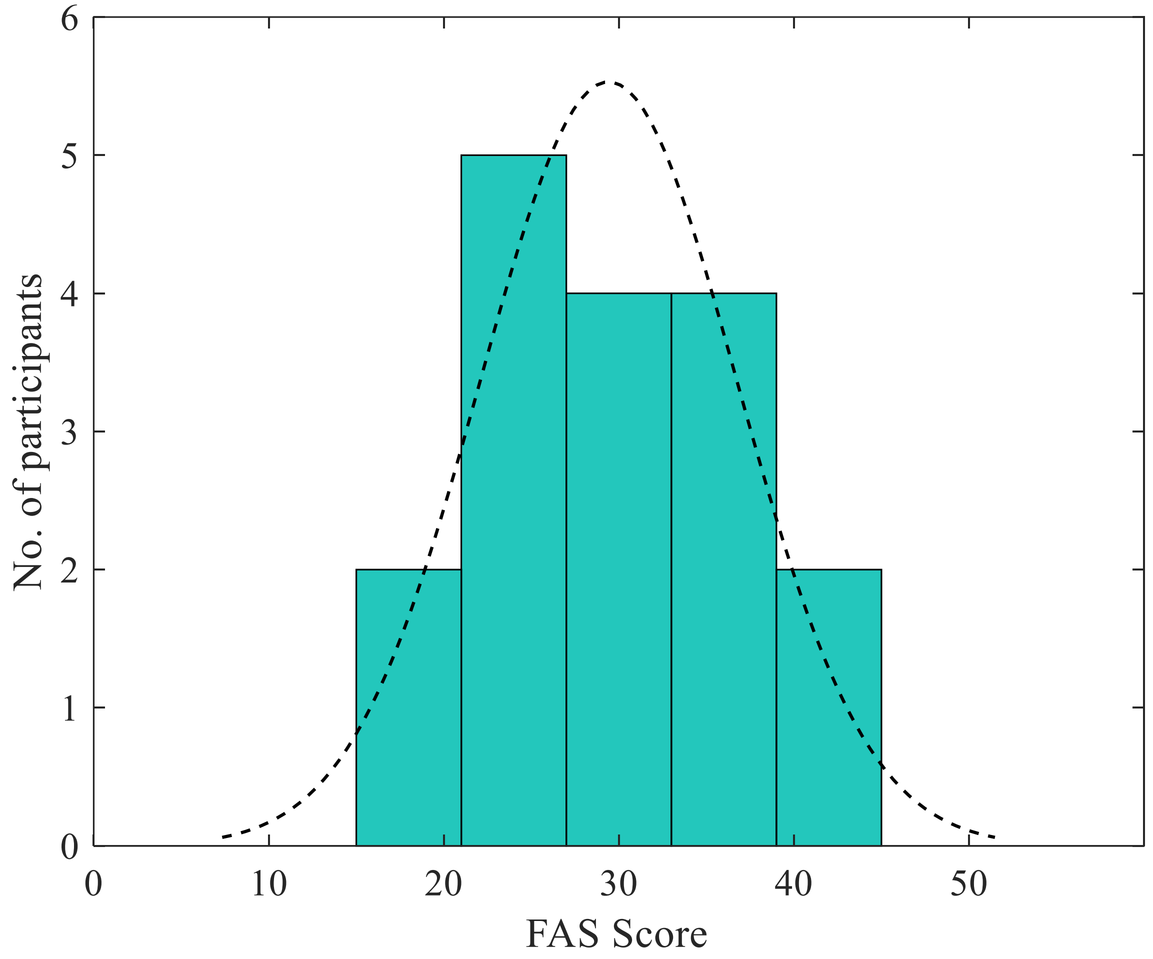

Figure S2. Distribution of FAS Scores obtained for all 17 participants during the Fatigue Assessment stage.

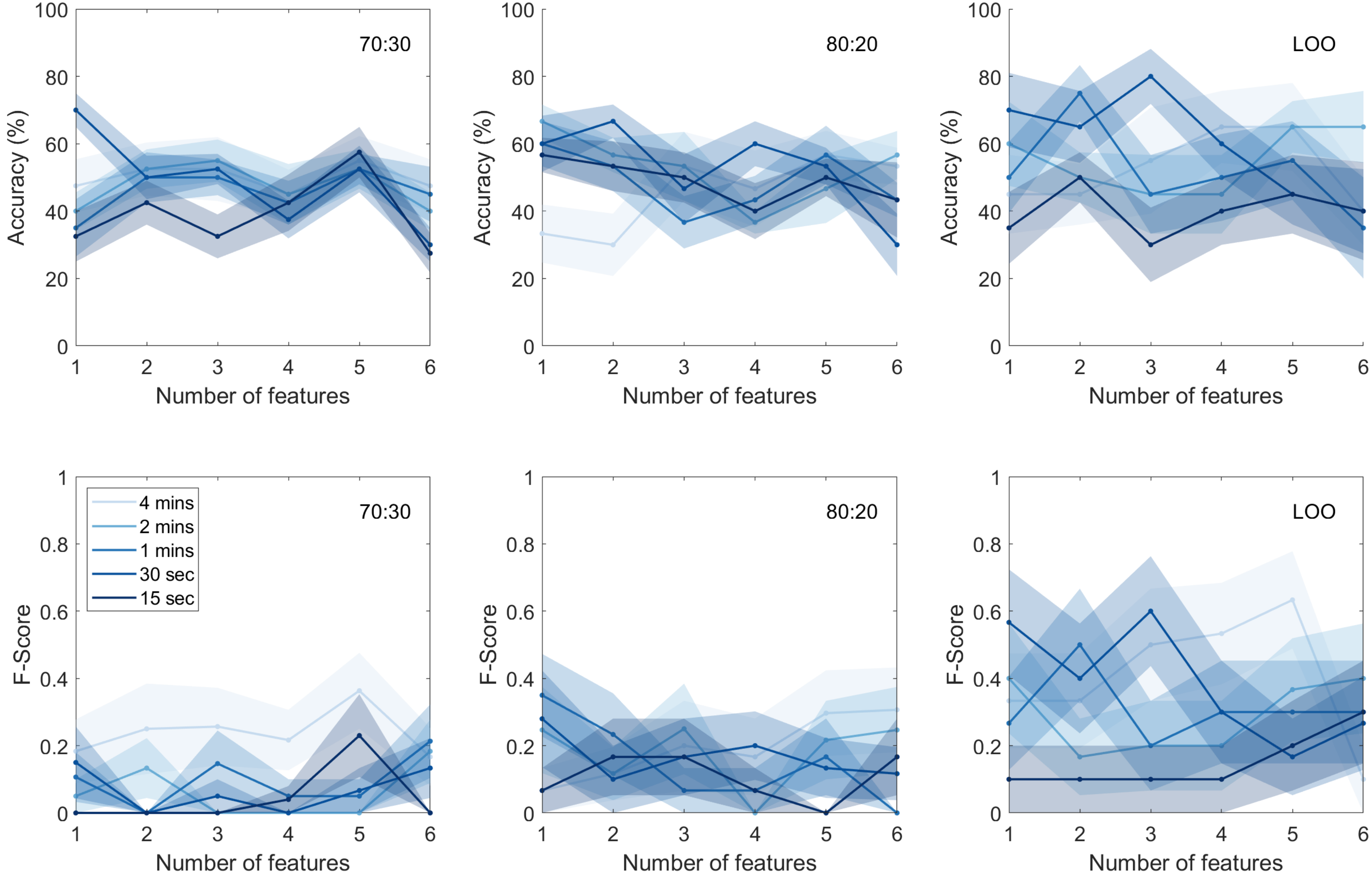

Figure S3. Average classification accuracies (top) and F-Scores (bottom) for the E4 model (K = 6 features) using 4, 2, 1 (min), 30 and 15 (sec) of data during the AO task. Shaded area represents standard error across cross-validations. Model performance was evaluated using 70:30 (left), 80:20 (middle) and LOO (right) data splitting approaches.

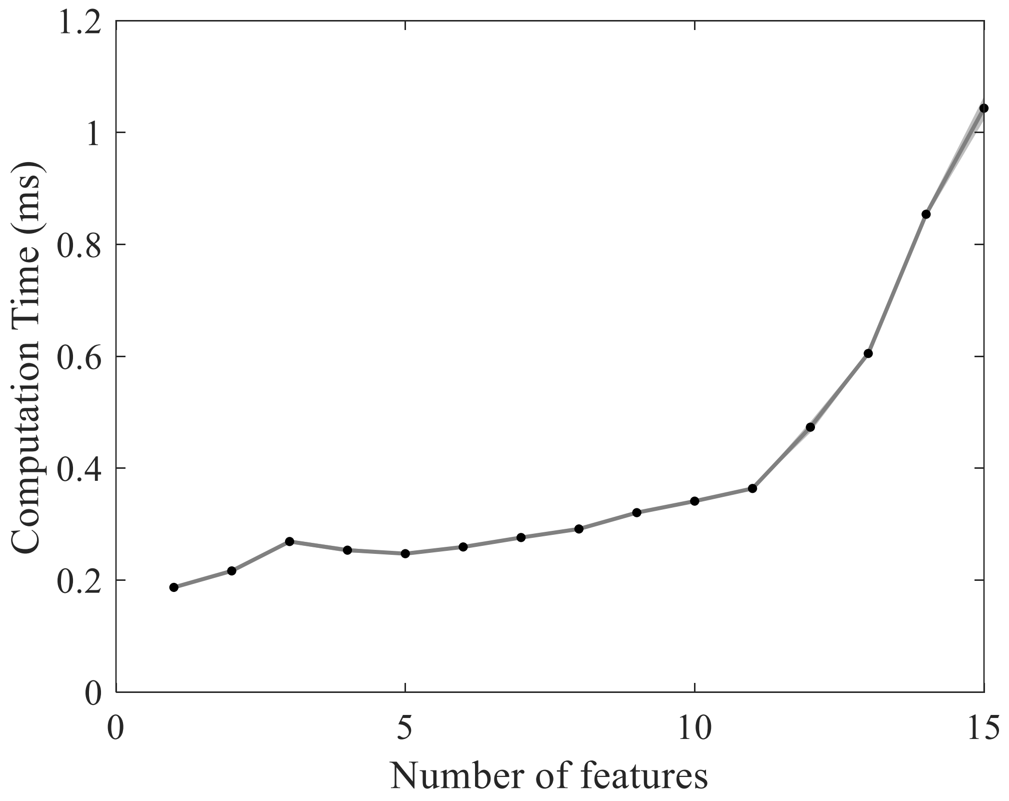

Figure S4. Average computation time needed to real-time make predictions using the one-minute (most accurate) EEG model. One hundred simulations were run, and the shaded area represents the standard error across the simulations.
